# Case Report: Coexistence of generalized arterial calcification of infancy (GACI) and maternal infections with cytomegalovirus and *Toxoplasma gondii*-unexpected fatal complication in a newborn

**DOI:** 10.3389/fped.2022.922379

**Published:** 2022-08-18

**Authors:** Simona Gurzu, Diana Burlacu, Réka Sánta, Ioan Jung, Mark Slevin, Emöke Fulop

**Affiliations:** ^1^Department of Pathology, George Emil Palade University of Medicine, Pharmacy, Science and Technology, Târgu Mureş, Romania; ^2^Department of Pathology, Clinical County Emergency Hospital, Târgu Mureş, Romania; ^3^Research Center in Oncology and Translational Medicine (CCOMT), University of Medicine, Pharmacy, Science and Technology, Târgu Mureş, Romania; ^4^Department of Pediatrics and Neonatology, Clinical County Emergency Hospital, Târgu Mureş, Romania; ^5^Department of Pediatrics, George Emil Palade University of Medicine, Pharmacy, Science and Technology, Târgu Mureş, Romania; ^6^Doctoral School, George Emil Palade University of Medicine, Pharmacy, Science and Technology of Târgu Mures, Târgu Mures, Romania

**Keywords:** vascular calcification, neonatal prematurity, autopsy, toxoplasmosis, histology

## Abstract

Generalized arterial calcification of infancy (GACI) is an extremely rare autosomal recessive condition characterized by the storage of calcium at the level of internal elastic membrane of arteries. The main consequences are intimal fibrous thickening and arterial occlusion. We present the case of a preterm male infant, born from an improperly dispensed pregnancy. At birth, the newborn presented generalized edema and hypotonia, and abolished heart sounds, without response to stimulation. Despite the mechanical ventilation, the infant died 2 h after birth. The death was clinically presumed to be related to the maternal infection with cytomegalovirus (CMV) and *Toxoplasma gondii*. The infant's mother affirmed the history of 6 previous miscarriages and a non-consanguineous marriage. At autopsy, microscopic examination showed generalized vasculitis secondary to minimal calcification of the large and medium-sized vessels of the lungs, liver, and tongue. These findings supported the diagnosis of GACI. Hydrothorax, non-infective ascites, and necrosis of the brain parenchyma were also associated. The premature infant died due to tonsillar herniation associated with decreased vessel compliance and refractory pulmonary hypertension thus leading to congestive cardiac failure. CMV was not detected on histopathological assessment nor were signs of any other infections. To the best of our knowledge, this is the first case of GACI occurring in a baby from a mother co-infected with CMV and *T. gondii*.

## Introduction

Bryant and White first described the generalized arterial calcification of infancy (GACI) in 1901 after performing the autopsy of a 6-months-old child (1). It is a rare fatal disease that presents in early infancy (2). With 50 cases reported before 1971 (3) and another 150 reported worldwide since then (4–7), it occurs with an estimated frequency of 1:390,000 infants (2).

GACI is considered idiopathic or a metastatic calcification of the large and medium-sized arteries secondary to advanced renal diseases, anomalies of the cardiovascular system, hypervitaminosis D, or parathyroid abnormalities (4, 8–13). Most recent theories refer to GACI as a genetic disease that might be caused by mutations in the ectonucleotide pyrophosphatase/phosphodiesterase 1 gene (ENPP1). ENPP1 gene is known to inhibit the storage of calcium at the level of soft tissues. Its mutation is inherited in an autosomal recessive pattern and is mostly seen in relation to consanguinity (4, 8–10).

At autopsy, GACI was reported in infants between 4 days and 20 months after birth (3–7). In this paper, we present the fatal case of the youngest newborn, which was reported in Medline literature, with non-consanguinity related GACI and the first instance in which the mother had a co-infection with CMV and *T. gondii*.

## Case report

### Clinical history

A 42-year-old, gravida 13, para 7, non-consanguineous married pregnant woman, in her 32nd week of pregnancy, was admitted to the Emergency Unit for polyhydramnios and pre-labor rupture of membranes (PROM). She declared an improperly dispensed pregnancy, with no regular gynecological consultations. She also affirmed that she had experienced 6 previous miscarriages.

Emergency cesarean section was performed and a male fetus was prematurely delivered. His Appearance, Pulse, Grimace, Activity, and Respiration (APGAR) scores were 1 at 1 min, 2 at 5 min, and 3 at 10-, 15-, and 20 min. At birth, the newborn presented generalized edema, cyanotic skin with petechiae, and generalized hypotonia, bradycardia, abolished heart sounds, without response to stimulation. As no spontaneous respiration occurred immediately after birth, endotracheal intubation, mechanical ventilation as well as continuous positive inotrope support was provided. Ultrasound examination revealed bilateral pleural effusion, ascites, and pericardial effusion. Despite the aggressive treatment, 2 h after being delivered, the infant died and an autopsy was requested. The mother granted her signed consent for the autopsy to be performed.

### Maternal investigations

Intrauterine infection was detected and serum examinations were performed, in order to rule out Toxoplasmosis, Other agents, Rubella, Cytomegalovirus, and Herpes Simplex (TORCH) syndrome. The paraclinical examinations indicated normal ranges for anti-hepatitis B and C and anti-rubella antibodies, same as for Immunoglobulin M (IgM) anti-CMV, anti-rubella, and anti-*Toxoplasma gondii*. High serum values were detected for anti-CMV Immunoglobulin G (IgG) (414.5 U/mL; normal range 0–0.5 U/mL) and anti-*T. gondii* IgG (55.30 U/mL; normal range 0–1 U/mL). All the biochemical tests, for detection of IgG/IgM, were performed through electrochemiluminescent immunoassay using Cobas e 411 analyzer and the diagnostic kits from the same manufacturer (Roche Diagnostics GmbH, Mannheim, Germany).

### Autopsy findings

**Figure 1:**
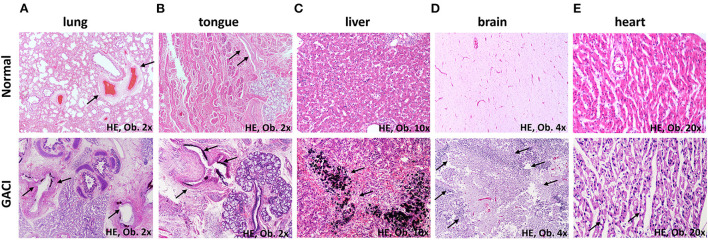
Microscopic findings of the multisystemic GACI compared with normal structures (arrows): **(A)** Calcifications of the medium-sized arteries of the lung. **(B)** Intimal calcifications of the arteries of the tongue. **(C)** Calcifications of the hepatocytes. **(D)** Necrosis of the brain parenchyma. **(E)** Hydropic changes of the myocardial fibers.

The 2,850 g preterm baby presented cyanotic skin and generalized edema, with hydrothorax, non-infective ascites, and hydrocele. The heart and the large vessels were macroscopically unremarkable. Protein dystrophy was seen in the myocardial fibers (Figure 1), without signs of myocardial ischemia or histological modifications of the coronary arteries.

**Figure 2:**
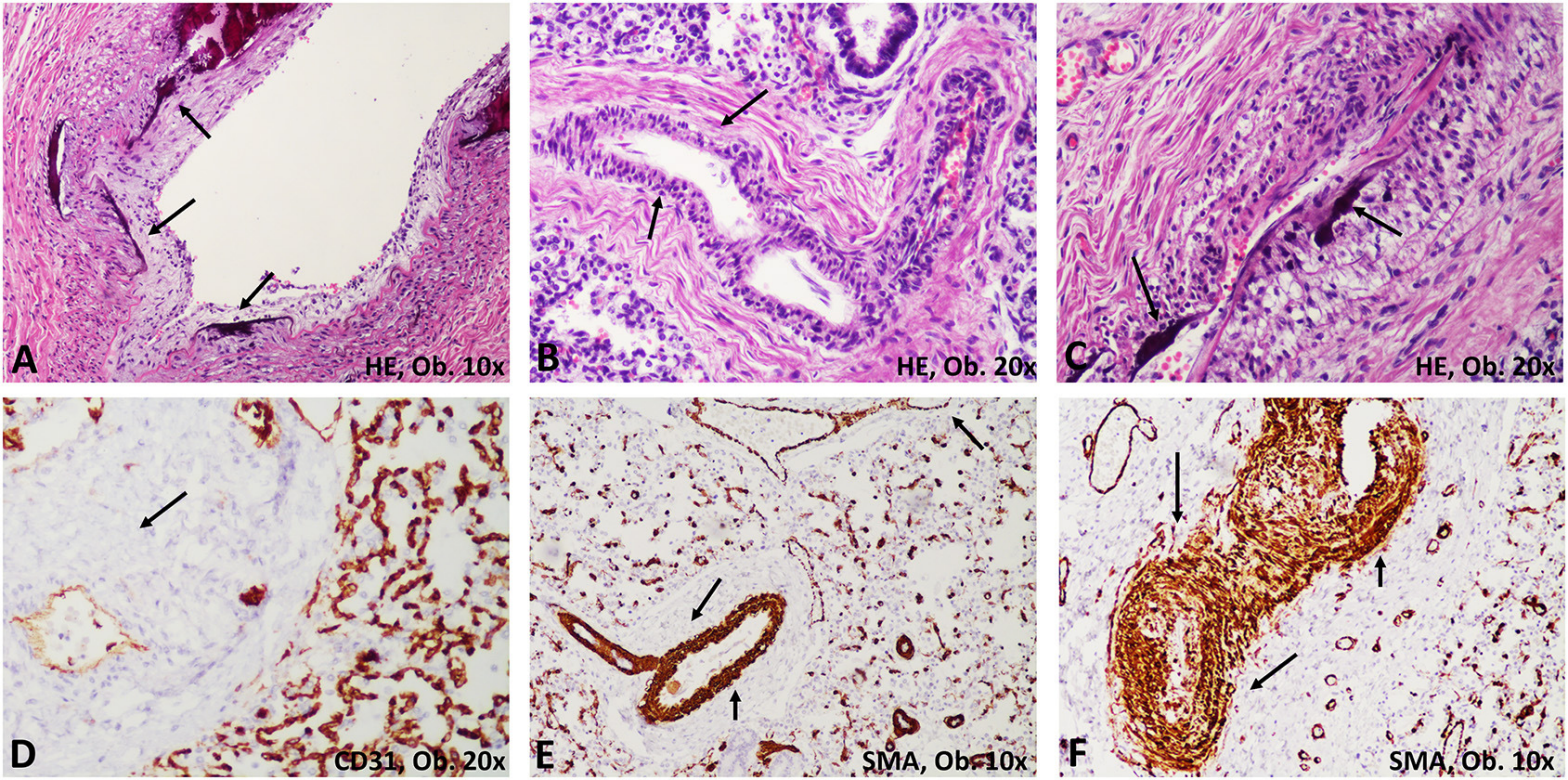
Histological modifications of the pulmonary arteries (arrows). **(A)** Calcifications of the lamina elastica; **(B,C)** Thickening of the intima with calcifications and stenosis of the arterial lumen. **(D)** The proliferated cells are negative for CD31. **(E,F)** Compared with normal arteries, marked proliferation of myofibroblasts is emphasized with smooth muscle actin (SMA).

Bilateral dystelectasis of the lungs was macroscopically described. Microscopic examination of the lung parenchyma revealed a predominance of vascular abnormalities, without pulmonary edema, hyaline membranes, bronchopneumonia, or other modifications of the lung parenchyma. The intima of large and medium-sized arteries showed marked thickness with degenerative calcifications of lamina elastica interna (Figure 1). In the intima, a proliferation of myofibroblasts that expressed Smooth Muscle Actin (SMA) and Cluster of Differentiation 34 (CD34) was predominant, without proliferation of Cluster of Differentiation 31 (CD31) -marked endothelial cells (Figure 2). Myointimal calcifications at the level of the hepatic vessels associated with necrosis of the perivascular hepatocytes (Figure 1).

At the base of the tongue and in the lower third of the pharynx, hemorrhagic areas of 7–12 mm were seen. Microscopically, the calcification of the intimal layer of the arteries was identified as similar to that of the pulmonary vessels (Figure 1).

Cerebral edema, tonsillar herniation, and extensive areas of necrosis were seen in the brain parenchyma (Figure 1). Death was identified as a result of acute respiratory failure, which was mainly induced by compression of the central respiratory centers and the external compression of the lung tissue leading to persistent pulmonary hypertension and heart failure.

No histological signs of CMV (e.g., ductular proliferation or cholestatis in the liver; the presence of giant cells; cytopathic changes; CMV inclusions) were seen and no immunohistochemical (IHC) positivity for CMV was proved in any of the examined organs (brain, kidney, spleen, lymph nodes, liver, lungs) (14). No neuronal degeneration or leukomalacia, as indicators of toxoplasmosis (15), was detected in the tissues harvested at autopsy.

## Discussion

GACI is an unusual and uncommon cause of death among newborns. This is the only case in our university hospital that has been seen in over a decade. Over the same period, almost 200 autopsies were performed annually, one-third of which were autopsies of neonates.

As a non-usual disease, only limited data about the microscopic aspects of GACI have been gathered. GACI is defined by marked calcification of the internal elastic membrane of the large and medium-sized arteries, fibroblastic proliferation in the intima, and a giant cell reaction (3, 6, 11). Coronary arteries were mostly described to be narrowed, with ventricular dysfunction or myocardial infarction as the major complications (7, 11, 16, 17). In our case, no signs of myocardial ischemia, myocarditis, nor foci of calcification were seen in the coronaries. Also, no giant cells were identified. Myointimal calcifications of the walls of the arteries were seen in the lung, liver, and base of the tongue. The proliferated cells proved to be myofibroblasts which were marked by CD34 and SMA but not by CD31 (18). No other cases with tongue involvement were reported till now.

Although necrosis of the hepatocytes and brain parenchyma might be related to CMV or *Toxoplasma* infection of the mother, previous data denied any possible connection between GACI and viral infections such as Coxsackie B viremia (13). In our case, we were also not able to prove the existence of encephalitis caused by a primary infection in the mother. In a previously published article, neurologic manifestations were reported in patients with GACI, with magnetic resonance imaging (MRI) proved strokes, gliosis, bilateral occipital necrosis, and cystic encephalomalacia (2) but no histopathological findings were shown. As miscarriage, hydrops, polyhydramnios, and fetal distress were seen in our and other reported cases (3, 19), it can be supposed that GACI might be a favoring factor rather than a consequence of intrauterine infections. On the other hand, in the present case, the mother's paraclinical tests indicated high serum values for anti-CMV IgG and anti-*T. gondii* IgG, but not for the IgM. This data indicated a chronic maternal infection that was probably not transmitted to the fetus during pregnancy, as the histological examination of the tissues harvested from the autopsy confirmed.

Although GACI has long been considered an idiopathic disease, the most recent case reports showed a close relation between ENPP1 gene mutations and consanguinity (13, 20). In our case, the infant was born from a non-consanguineously married woman, who indeed suffered from multiple unexplained miscarriages. Genetic investigations were not requested by the mother.

Untreated, death of the infant occurs before the age of 6 months by myocardial ischemia and cardiorespiratory arrest (6, 19). In any baby with pulmonary hypertension and hydramnios, GACI should be suspected (6). When diagnosed early, by fetal ultrasound, calcium chelator therapy with sodium thiosulfate or other bisphosphonates that solubilize calcium depositions, along with magnesium supplementation might prolong life until adolescence or early adulthood (2, 6, 17, 19). Although no curative therapy exists, extracorporeal membrane oxygenation along with supportive therapy might be the preferred therapy of choice (19).

The present unusual case was presented to highlight the need to more attentively evaluate patients with TORCH syndrome. Even if neurological or cardiovascular disorders might be seen in such infants, GACI should be considered a possibility.

## Data availability statement

The raw data supporting the conclusions of this article will be made available by the authors, without undue reservation.

## Ethics statement

Ethical review and approval was not required for the study on human participants in accordance with the local legislation and institutional requirements. Written informed consent was obtained from the newborn mother to perform the autopsy and publish the scientific results.

## Author contributions

SG drafted the paper and performed the design of the study. DB performed the autopsy and contributed to the case description, histopathological assessment, and literature review. IJ and EF contributed to histopathological and immunohistochemical assessment. RS performed the clinical management of the case and interpretation of the clinical data. MS participated at the design of the project and funding of the study. EF participated at the autopsy, contributed to macroscopic, and conferred the final agreement for publication. All authors contributed to the article and approved the submitted version.

## Funding

This study was partially funded by the CNCS - UEFISCDI, project number PN-III-P4-ID-PCE2020-1540 - Director of Project MS. The English proofread was done by Cambridge Proofreading LLC Professional Team.

## Conflict of interest

The authors declare that the research was conducted in the absence of any commercial or financial relationships that could be construed as a potential conflict of interest.

## Publisher's note

All claims expressed in this article are solely those of the authors and do not necessarily represent those of their affiliated organizations, or those of the publisher, the editors and the reviewers. Any product that may be evaluated in this article, or claim that may be made by its manufacturer, is not guaranteed or endorsed by the publisher.

## Abbreviations

APGAR: Appearance, Pulse, Grimace, Activity and Respiration
CD: Cluster of Differentiation
CMV: Cytomegalovirus
ENPP1: Ectonucleotide pyrophosphatase/phosphodiesterase 1
GACI: Generalized arterial calcification of infancy
Ig: Immunoglobulin
IHC: Immunohistochemistry
MRI: Magnetic Resonance Imaging
PROM: Pre-labor rupture of membranes
SMA: Smooth Muscle Actin
TORCH: Toxoplasmosis, Other agents, Rubella, Cytomegalovirus, Herpes Simplex.
